# A few steps closer to answering the unanswered questions about higher calorie refeeding

**DOI:** 10.1186/s40337-017-0139-1

**Published:** 2017-03-01

**Authors:** Andrea K. Garber

**Affiliations:** Department of Pediatrics, Division of Adolescent and Young Adult Medicine, University of California, San Francisco, Benioff Children’s Hospital, 3333 California St., Suite 245, Box 0503, San Francisco, CA 94118 USA

**Keywords:** Anorexia nervosa, Eating disorder, Refeeding, High calorie, Low calorie, Safety, Nutritional rehabilitation, Refeeding syndrome

Refeeding is the first step in long-term nutritional rehabilitation of patients with anorexia nervosa (AN). It may begin in the outpatient setting or in the hospital, if close medical and/or psychiatric monitoring is needed. In either case, early weight gain appears to be crucial for recovery. In hospital, faster weight gain [1] and higher weight upon discharge [2, 3] predict weight recovery at 1 year. In outpatient psychotherapy, substantial early gain (at least 0.43–0.86 kg/week during the first 4 weeks) predicts both weight and cognitive recovery at 12 months [4]. The importance of weight gain, however, must be balanced with the need for safety. Around the time of World War II, deaths were documented during refeeding in prisoners of war and the refeeding syndrome was first described [5, 6]. These sobering reports launched a 60-year period of extremely conservative approaches, with consensus-based recommendations for AN beginning around 1200 cal per day (kcal/d) and advancing slowly by 200 kcal every other day [7–9]. During the decades that this “start low and go slow” approach has been the standard of care in AN, only a handful of cases of the refeeding syndrome have been reported and this has been lauded as an indication of the safety. However, recent studies linking LCR to poor weight gain [10–12] and prolonged hospital stays [11, 12] have contributed to recognition of the “underfeeding syndrome” [13] and spurred interest in Higher Calorie Refeeding (HCR).

There is no consensus as of yet on the definition of HCR, and clinical approaches vary widely. Nevertheless, it is clearly a “hot topic”: 85% of the refeeding studies published from 2010 to 2015 started with higher calories than recommended (≥1400 kcal/d) [14]. This issue of the *Journal of Eating Disorders* is on the frontline of this movement. The studies published in this issue share core attributes: all three are retrospective chart reviews examining short-term, meal-based refeeding in moderately malnourished [75–85% of median Body Mass Index (mBMI)] adolescents (ages 14–16 on average), who were diagnosed with eating disorders and hospitalized for medical stabilization. However, several unique features further our understanding by addressing important unanswered questions.

**Is HCR safe?** No study to date has established the safety of a refeeding approach, and perhaps no study could ever truly do so, since a massive study population would be required to examine the full spectrum of features of the rarely occurring refeeding syndrome. Thus, we must continue to use very careful language around safety, acknowledging that electrolyte shifts are at best only early indicators of the risk for development of the refeeding syndrome. This was the focus of **Maginot et al.**, who compared serum phosphorus, potassium and magnesium during refeeding on LCR (starting around 1185 kcal/d and advancing by about 90 kcal/d) versus HCR (starting at 1781 kcal/d on average and advancing by about 100 kcal/d). Among 89 participants, the authors found no association between starting kcal prescription and hypophosphatemia, hypokalemia or hypomagnesaemia in the first 72 h. Another unique aspect of this study was its subanalysis of severely malnourished patients: almost 30% of the study population was admitted with mBMI < 75%. Only two prior studies of HCR have had sufficient sample sizes to do this (as reviewed in ref 14), both reporting higher rates of refeeding hypophosphatemia in such patients. Maginot et al.s’ finding that admission %mBMI (not kcal prescription) was the best predictor of hypophosphatemia is consistent with the current evidence [15] and warrants continued caution when refeeding severely malnourished patients.

**Is HCR appropriate for all eating disorder patients?** The 5^th^ Edition of the Diagnostic and Statistical Manual (DSM-5) has improved the accuracy and broadened the range of eating disorder diagnoses [16]. This shift is reflected in the hospitalized population: more than one-third of the 215 participants in the **Peebles et al.** study were diagnosed with eating disorders other than AN, including Bulimia Nervosa (BN), Avoidant and Restrictive Food Intake Disorder (ARFID)﻿, Atypical AN (AAN), Purging Disorder or Unspecified Food and Eating Disorder (UFED). This study took a unique quality improvement perspective, providing a detailed description of a multi-system refeeding approach including parents. Starting with an average of 1466 kcal and advancing by 233 kcal/d, all groups gained significant weight during the average 11 day hospital stay and this was sustained in the 74% of participants who followed-up at 4 weeks. This finding demonstrates that the program’s approach to refeeding is consistent with their non- weight-biased belief that all patients who are medically compromised to the point of meeting hospital admission criteria should gain initial weight, regardless of specific diagnosis.

**Does HCR lead to better outcomes?** Relapse in the first year of recovery is common: up to 43% of patients will require medical rehospitalization within 12 months of discharge [17]. Practitioners have voiced understandable concerns that the shorter hospitalizations associated with rapid refeeding could create a “revolving door” of frequent rehospitalizations. The study by **Smith et al.** is the first to examine the relationship between the caloric level of refeeding and outcomes beyond the hospital stay. Participants were refed beginning around 1585 kcal/d and advanced variably to achieve a weight regain of 130–200 g per day (average advancement was 146 kcal/d). In 4 weeks, 70% of the 129 participants followed-up and 9% were readmitted. In growth models, faster caloric advancement was associated with greater weight gain in hospital but not weight at 4-week follow-up or readmission. While this finding may disappoint those who hoped that HCR would produce better post-hospital outcomes, it should be viewed as a positive sign that equally good mid-term recovery may be achieved over a shorter hospital stay using HCR.

**Future directions –** The studies highlighted here break new ground and point to several priority areas for future studies of refeeding. The first priority must be safety. Fortunately, no study of HCR to date (including the three here) has reported a case of the refeeding syndrome. However, this should not be misinterpreted to mean that HCR carries no risk. In fact, about half of the participants in both the Maginot et al. and Smith et al. studies developed hypophosphatemia. Thus, the collective research supports the feasibility of HCR in the highly controlled hospital setting with close medical monitoring and electrolyte replacement. Even within these settings, wide variations in approaches to care may explain disparate findings, such as the 77% of the participants in Maginot et al. who required electrolyte correction, compared to only 14.2% in Peebles et al. A second priority for future investigations is to follow the example of the present studies and describe feeding protocols in detail (Peebles et al. set the high bar here). Two-thirds of “refeeding” studies published from 2010 to 2015 failed to provide basic information such as caloric level [14], which hampers interpretation and reproducibility of findings. Third, future studies should further extend the 4-week follow-up period of Smith et al. and Peebles et al. to examine longer-term outcomes of refeeding. Any such proposals would be well supported by a large scale Randomized Controlled Trial (RCT) showing that long hospital stays do not improve 1-year recovery rates in AN [18]. On that note, all of these research priorities could be simultaneously addressed with well-designed RCTs that directly compare approaches to feeding. RCTs would also address the problem of selection bias, whereby physicians tend to prescribe LCR to more malnourished patients. This problem has plagued our own work [10, 11] and was likely at play in Maginot et al., where the LCR group tended to have lower admit weight than the HCR group. Without randomization, such bias is nearly unavoidable, since the decision to exercise caution with more malnourished patients is indeed evidenced-based [15]. The tremendous amount of time and resources that such RCTs will require may seem daunting, but to skip this crucial step in scientific inquiry and adopt HCR as the new standard of care for AN would be to risk embarking on another 65 years of consensus-based (rather than evidence-based) care. Our patients deserve more! Bravo to the authors highlighted here for paving the way.

## Abbreviations

AN: Anorexia nervosa
HCR: Higher calorie refeeding
LCR: Lower Calorie Refeeding
mBMI: median Body Mass Index
RCT: Randomized Controlled Trial
AN: Anorexia Nervosa
